# Spectral–Structural Collaborative Learning for Fine-Grained Hyperspectral Mineral Classification

**DOI:** 10.3390/s26144345

**Published:** 2026-07-09

**Authors:** Yichun Qiu, Yanshuang Zhang, Shixian Cao, Wenyuan Wu, Shanjuan Xie

**Affiliations:** 1 School of Information Science and Technology, Hangzhou Normal University, Hangzhou 311121, China; 2023213101053@stu.hznu.edu.cn (Y.Q.); caoshixian@stu.hznu.edu.cn (S.C.); wuwy@hznu.edu.cn (W.W.); 2 Kharkiv College, Hangzhou Normal University, Hangzhou 311121, China; 3 College of Artificial Intelligence and E-Commerce, Zhejiang Gongshang University Hangzhou College of Commerce, Hangzhou 311121, China; zys@zjhzcc.edu.cn; 4 Zhejiang Provincial Key Laboratory of Urban Wetlands and Regional Change, Hangzhou Normal University, Hangzhou 311121, China

**Keywords:** hyperspectral image classification, fine-grained hyperspectral mineral classification, deep learning, collaborative attention paradigm

## Abstract

Fine-grained hyperspectral mineral classification remains challenging due to spectral homogeneity among minerals with different morphologies, severe spectral mixing from intergrowth, and high dimensionality. Existing methods rely on spectral separability assumptions, which become insufficient when spectral differences are subtle and spatial–structural ambiguity is high. To address these limitations, we propose S^3^AM-ECA-3DCNN, a spectral–structural collaborative feature learning framework. It uses a 3DCNN backbone to jointly model spectral–spatial features with progressive spectral downsampling. The spectral-similarity-based spatial attention module (S^3^AM) performs spatial purification by suppressing interference from spectrally mixed neighboring regions, and the efficient channel attention (ECA) module adaptively recalibrates discriminative spectral bands to enhance fine-grained representation. This establishes a spatial-first, channel-second collaborative optimization paradigm. To improve generalization under limited training samples, adaptive global pooling and a lightweight classification head are employed to reduce model complexity and mitigate overfitting. Experiments on a 146-class hyperspectral mineral dataset (covering silicates, carbonates, and sulfates) show that the framework achieves 93.424% overall accuracy, 91.099% average accuracy, and a Kappa coefficient (×100) of 93.368, outperforming mainstream methods. It significantly reduces misclassification among spectrally similar but morphologically distinct minerals, demonstrating strong robustness and discriminative capability for large-scale fine-grained tasks.

## 1. Introduction

Mineral identification plays a critical role in geological exploration, ore processing, and mineral resource management, directly affecting resource utilization efficiency and industrial sustainability [1,2]. With the increasing demand for automated and non-destructive mineral analysis, hyperspectral imaging (HSI) has emerged as an effective technique for mineral characterization owing to its capability to simultaneously capture spatial and spectral information [3,4,5,6,7].

Early studies relied on traditional machine learning methods (e.g., logistic regression, random forests, SVM) [8,9,10,11,12,13,14,15,16]. Recent years have witnessed significant advances in close-range laboratory hyperspectral imaging for mineral analysis [17,18,19]. However, conventional machine learning methods are often limited by the curse of dimensionality and their insufficient ability to model spatial contextual information in high-dimensional hyperspectral data [20].

Deep learning has significantly advanced hyperspectral image classification by enabling end-to-end feature learning [21,22]. CNNs have demonstrated strong performance in hyperspectral and rock image analysis through automatic feature extraction [23,24,25], while subsequent studies have explored ensemble learning strategies [26], parameter-efficient DenseNet architectures [27], multi-scale ResNet architectures for feature extraction [28,29], and an AdaBoost-enhanced convolutional recurrent neural network for fine-grained hyperspectral lithological classification [30]. Among these, 3DCNN architectures [31] have been widely adopted because they jointly exploit spectral and spatial information through volumetric convolutions. Nevertheless, most existing deep learning methods still rely primarily on spectral separability, which is often inadequate for fine-grained mineral classification, where minerals with different morphologies, aggregate structures, or crystallization states may exhibit highly similar spectral signatures.

In practical geological environments, mineral classification is fundamentally challenged by spectral–structural ambiguity. Specifically, minerals with distinct morphologies or aggregate structures may share nearly identical spectral responses, while mineral intergrowth and boundary mixing further distort discriminative spectral features [32,33]. Consequently, spectral similarity does not necessarily correspond to mineralogical identity, leading to severe confusion among fine-grained mineral categories.

This problem is particularly pronounced in large-scale mineral classification tasks involving numerous visually and spectrally similar categories. Under such conditions, existing CNN-based frameworks struggle to establish stable and discriminative feature representations [34]. Semantic-guided representations benefit fine-grained discrimination, as demonstrated by Li et al. [35] using C&D-CLIP for zero-shot segmentation. However, such RGB-based pretraining is not directly applicable to hyperspectral mineral classification, where spectral–spatial modeling is essential.

However, standard 3DCNNs remain limited in handling spectral mixing and subtle inter-class differences in fine-grained mineral classification. Attention mechanisms help models focus on discriminative spectral bands and spatial regions to better distinguish minerals with similar spectral responses. Recent efforts include channel attention in EdgeConv [36], evaluation of attention modules in ResNet [37], and the nonlocal multi-scale attention for capturing long-range spectral–spatial dependencies to discriminate similar materials [38].

Coordinating spatial and channel attention is increasingly recognized as beneficial for feature representation. CBAM [39] demonstrated the benefit of combining both, yet lacks domain-specific guidance for hyperspectral data. SSFTT [40] adapts Vision Transformers to HSI classification but incurs high computational cost. More relevantly, S^3^AM [41] provides spectrally guided spatial attention, while ECA [42] offers lightweight channel recalibration without dimensionality reduction. Despite their individual merits, no systematic integration of spectral-similarity-guided spatial attention with efficient channel attention has been specifically tailored for fine-grained mineral classification.

For fine-grained hyperspectral mineral classification, spatial purification should precede spectral recalibration. Spatial interference and boundary mixing directly contaminate spectral representations in early feature extraction stages; therefore, suppressing spatial ambiguity before channel-wise enhancement is essential for establishing reliable discriminative representations.

The main contributions of this study are summarized as follows:

(1) Spectral–structural ambiguity is identified as a fundamental bottleneck in fine-grained hyperspectral mineral classification, revealing the limitation of conventional spectral separability assumptions in complex geological scenarios.
(2) A spatial-first collaborative attention framework is introduced, integrating spectral-similarity-guided spatial purification with efficient spectral channel recalibration. This enables joint optimization of spatial localization and spectral discrimination.
(3) A bidirectional collaborative optimization mechanism between S^3^AM and ECA improves feature discriminability under severe spectral similarity and mineral intergrowth.
(4) Extensive experiments on a 146 class mineral dataset demonstrate that the proposed framework achieves superior accuracy, robustness, and generalization compared with mainstream hyperspectral classification methods.

To address spectral–structural ambiguity and feature discrimination limitations in fine-grained hyperspectral mineral classification, this study proposes S^3^AM–ECA–3DCNN, a spectral–structural collaborative feature learning framework. Built on a 3DCNN backbone with progressive spectral downsampling, it establishes a spatial-first collaborative optimization paradigm by serially coupling a spectral-similarity-based spatial attention module (S^3^AM) with an efficient channel attention (ECA) mechanism. S^3^AM suppresses spatial interference from mineral intergrowth and boundary mixing, while ECA performs adaptive spectral recalibration. This coordinated optimization enhances discriminative representation learning under spectral homogeneity, subtle inter-class variations, and limited samples. Extensive experiments on a 146-class mineral dataset demonstrate superior classification accuracy, robustness, and generalization over mainstream methods, confirming its effectiveness for large-scale fine-grained mineral discrimination.

The remainder of this paper is organized as follows. Section 2 describes the hyperspectral mineral dataset and acquisition. Section 3 presents the proposed S^3^AM–ECA–3DCNN framework and its collaborative optimization mechanism. Section 4 reports experimental settings, comparative analyses, and discussion. Section 5 concludes the paper and outlines future work.

## 2. Hardware and Dataset

### 2.1. Hyperspectral Imaging System

**Figure 1:**
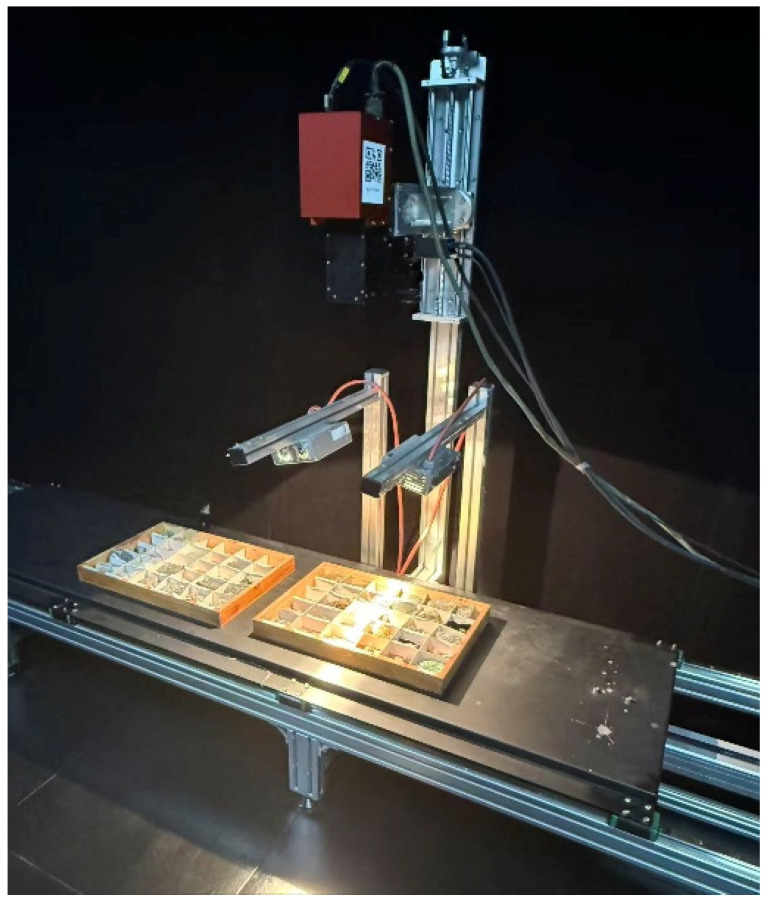
HySpex hyperspectral sensor.

This study employs the HySpex SWIR-384 push-broom hyperspectral imaging system (as shown in Figure 1). The system is mounted on a push-broom mobile platform, featuring a 16-degree field of view, a spatial resolution of 384 × 288 pixels, a spectral range of 950–2500 nm (with a spectral resolution of 5.45 nm), and 16-bit data precision. Equipped with an autofocus function, it enables high-precision multispectral imaging in dynamic scanning scenarios.

### 2.2. Hyperspectral Mineral Standard Dataset (HMSD-1.0)

**Figure 2:**
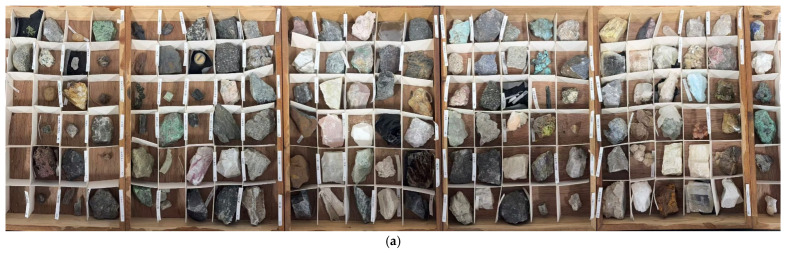

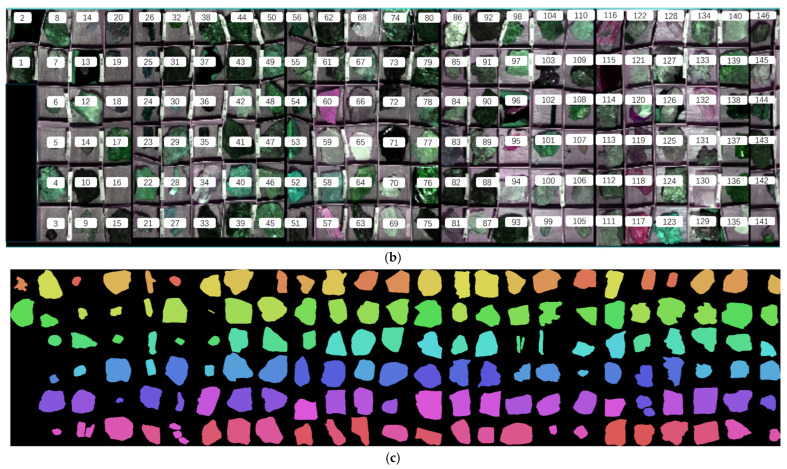
Dataset HMSD-1.0. (**a**) Classification of 146 mineral samples, (**b**) false color composite image of samples, and (**c**) true labels after corrosion.

The HMSD-1.0 dataset follows the International Mineralogical Association (IMA) crystal chemical classification [43] and includes 146 mineral classes, covering major groups such as silicates, carbonates, and sulfates. Representative samples include feldspar, calcite, beryl, and gypsum (see Figure 2a for classification display).

In the collection of mineral spectral data for this study, whiteboard calibration technology was employed to suppress sensor noise and environmental interference, thereby obtaining true reflectance data. Using a standard whiteboard with a reflectance close to 100% as the benchmark, the spectral reflectance was calculated according to Equation (1):

(1) $$R=\frac{H\cdot R_{w}}{E_{w}}$$

where H represents the hyperspectral emissivity image (raw sensor data); $R_{w}$ denotes the pre-calibrated reflectance of the white reference board; and $E_{w}$ refers to the white reference radiance, which is primarily used to normalize the sensor emissivity measurements for accurate surface reflectance.

The corrected hyperspectral pseudo-color image is shown in Figure 2b. This image effectively enhances the signal-to-noise ratio of the original data, and such a visualization method can directly reflect the spectral absorption characteristics of different mineral types. To address issues of edge blurring and shadow interference in mineral sample images, this study innovatively adopts a 5×5 erosion algorithm for morphological processing of sample edges. A comparison of the processing effect is provided in Figure 2c. This method effectively eliminates the impact of abnormal boundary pixels on spectral feature extraction, thereby improving the accuracy of the classification model.

## 3. Methodology

**Figure 3:**
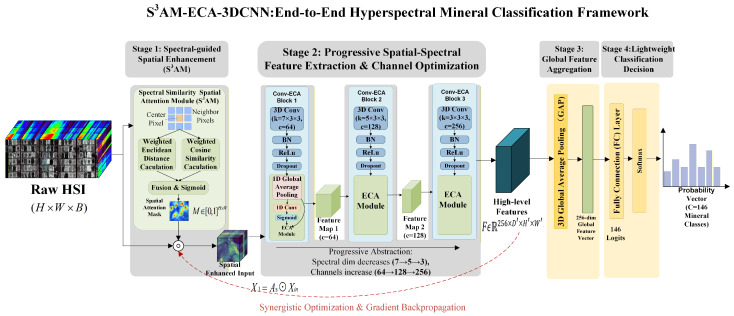
S^3^AM-ECA-3DCNN: End-to-end hyperspectral fine-grained mineral classification framework.

To address strong neighborhood interference, overfitting caused by high-dimensional hyperspectral data, and subtle inter-class spectral variations in fine-grained mineral classification, this study proposes a spectral–spatial deep collaborative feature learning framework. A 3DCNN backbone jointly models spectral–spatial information via progressive spectral downsampling, which aggregates informative spectral cues while preserving local spatial structures. A serial collaborative optimization mechanism deeply integrates an S^3^AM and an ECA module: S^3^AM first exploits spectral similarity to guide spatial attention, enhancing discriminative regions; ECA then performs adaptive channel reweighting to highlight informative spectral bands. This design enables coordinated optimization of spatial localization and channel selection, leading to more discriminative representations. Finally, adaptive global pooling and lightweight fully connected (FC) layers are used for classification. Owing to its low parameter count and computational overhead, the proposed framework achieves high accuracy, robustness, and generalization under noise, illumination changes, and limited training samples (see Figure 3 for the schematic diagram).

### 3.1. Overall Architecture of S^3^AM-ECA-3DCNN

The framework applies spatial attention (S^3^AM) first, then channel attention (ECA). This order ensures that spatial interference from intergrown minerals is suppressed before channel-wise enhancement of diagnostic spectral features.

Let the input hyperspectral data cube be denoted as $X_{in}\in R^{H\times W\times B}$, where H, W, and B denote height, width, and number of spectral bands. S^3^AM generates a spatial attention map $A_{s}\in {\left[0,1\right]}^{H\times W}$, applied via:

(2) $$X_{1}=A_{s}⊙X_{in}$$

where $A_{s}$ is broadcast along the spectral dimension. The refined feature $X_{1}$ is then processed by a three-stage 3DCNN backbone with an ECA module after each stage. For stage i∈{1,2,3}:

(3) $$X_{i}=\varepsilon_{i}\left(Ci\left(X_{i-1}\right)\right)$$

where the convolutional block $C_{i}\left(\cdot \right)$ is defined as:

(4) $$C_{i}\left(\cdot \right)=Dropout\left(ReLU\left(BN\left(3{DConv}_{i}\left(\cdot \right)\right)\right)\right)$$

The dropout rate is set to 0.5 for all three stages and $\varepsilon_{i}\left(\cdot \right)$ denotes the ECA operation. The backbone outputs yields a latent feature $X_{3}\in R^{H^{\prime }\times W^{\prime }\times D^{\prime }\times C}$ (with C=256, and $D^{\prime }$ is the spectral depth after downsampling). Finally, the classification head $H\left(\cdot \right)$ (comprising global pooling and a single FC layer) yields the prediction:

(5) $$\hat{y}=f\left(X\right)=H\left({\tilde{X}}_{3}\right)$$

### 3.2. Spectral-Guided Spatial Attention: S^3^AM Module

The S^3^AM module computes spatial attention based on spectral similarity. For a patch $X\in R^{\omega \times \omega \times B}$ centered at $p_{c}$ with spatial size ω×ω, S^3^AM computes two weighted similarity metrics: Weighted Euclidean Distance (WED) for amplitude variance ($S_{e}$) and Weighted Cosine Distance (WCD) for spectral shape ($S_{c}$):

(6) $$S_{e}\left(p_{c},x\right)=\sqrt{\sum_{i=1}^{B}w_{i}\cdot (p_{c}^{i}-x^{i}{)}^{2}}$$

(7) $$S_{c}\left(p_{c},x\right)=\frac{\sum_{i=1}^{B}w_{i}\cdot p_{c}^{i}\cdot x^{i}}{\sqrt{\sum_{i=1}^{B}w_{i}\cdot (p_{c}^{i}{)}^{2}}\cdot \sqrt{\sum_{i=1}^{B}w_{i}\cdot (x^{i}{)}^{2}}}$$

Here, $w\in R^{B}$ is a learnable weight vector produced by a fully connected band calibration (FBC) layer from the central pixel’s spectrum to emphasize diagnostic bands. The two metrics are normalized to [0,1] and fused via a learnable coefficient α∈[0,1]:

(8) $$S=\alpha \cdot S_{e}+\left(1-\alpha \right)\cdot \left(1-S_{c}\right)$$

The fused distance S is then transformed into a spatial attention mask $A_{s}$ via the spectral-guided (SG) activation function:

(9) $$A_{s}=e^{-\beta S^{2}}$$

where β is a learnable scaling parameter. The final refined input is:

(10) $$X_{1}=A_{s}⊙X_{in}$$

**Figure 4:**
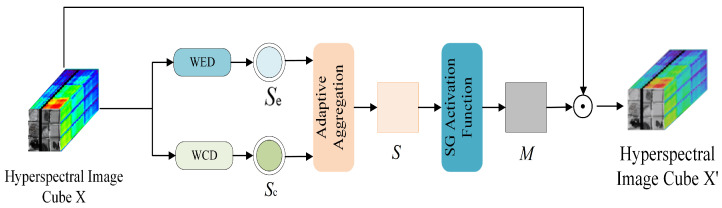
S^3^AM schematics. Where “⊙” denotes the element-wise multiplication.

Its specific structure is illustrated in Figure 4. To optimize fine-grained mineral classification, we introduce three modifications: (1) FBC initialization targets mineral-specific absorption bands (e.g., iron bands around 0.9 μm and hydroxyl bands around 2.2 μm); (2) SG parameters are tuned for complex spectral mixing; and (3) S^3^AM is positioned as the primary stage to ensure early interference suppression.

This design exploits the geological fact that compositionally similar minerals have correlated spectral responses: S^3^AM identifies coherent mineral regions via spectral similarity and suppresses interference from adjacent minerals with different signatures.

### 3.3. Channel-Spectral Co-Optimization: ECA Module

While S^3^AM addresses spatial localization, the ECA module performs adaptive weighting of spectral bands to emphasize diagnostic absorption features. Extending the original 2D-ECA [42] to 3D, we apply 3D global average pooling (GAP) over spectral–spatial dimensions. For an output tensor $X^{\left(l\right)}\in R^{H\times W\times D\times C}$ at layer l, GAP aggregates into a vector $z\in R^{C}$:

(11) $$z_{c}=\frac{1}{H\cdot W\cdot D}\sum_{i=1}^{H}\sum_{j=1}^{W}\sum_{k=1}^{D}x_{c}\left(i,j,k\right)$$

where D is the spectral depth after downsampling, and $x_{c}\left(i,j,k\right)$ is the element at channel c and spectral–spatial position (i,j,k).

Channel weights are generated via a 1D convolution with adaptive kernel size:

(12) $$k=\psi \left(C\right)=\;{\left|\frac{\log_{2}C}{\gamma }+\frac{b}{\gamma }\right|}_{odd}$$

where γ=2 and b=1 are hyperparameters, and $\;{\left|\cdot \right|}_{odd}$ denotes the nearest odd integer. The channel attention vector $A_{c}$ is obtained as:

(13) $$A_{c}=\sigma \left({Conv1D}_{k}\left(z\right)\right),\;\;A_{c}\in {\left[0,1\right]}^{C}$$

where σ(⋅) is the sigmoid function, and ${Conv1D}_{k}$ denotes a 1D convolution with kernel size k. ECA then recalibrates the original features via channel-wise multiplication:

(14) $${\tilde{X}}_{c}^{\left(l\right)}=A_{c}^{\left(l\right)}\cdot X_{c}^{\left(l\right)},\forall c\in \left\{1,\ldots ,C\right\}$$

where $A_{c}^{\left(l\right)}$ is the c−th element of the attention vector for the l layer.

**Figure 5:**
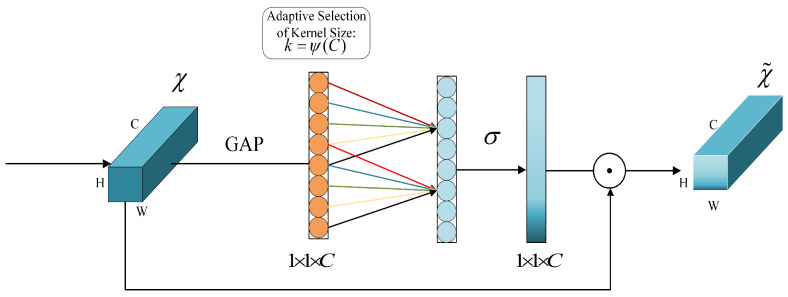
ECA module schematic diagram.

Its specific structure is illustrated in Figure 5. The parameter complexity of the ECA module is only O(k×C).

### 3.4. 3DCNN Backbone for Joint Feature Extraction

The 3DCNN backbone extracts joint spectral–spatial features via 3D convolutions. For an input feature map $x\in R^{H\times W\times D\times C}$, where C is the number of channels and H,W,D denote spatial height, width, and depth (or spectral bands), a 3D convolution computes the output value at position (i,j,k) as:

(15) $$y_{i,j,k}=\sum_{c=1}^{C}\sum_{p=0}^{P-1}\sum_{q=0}^{Q-1}\sum_{r=0}^{R-1}w_{c,p,q,r}\cdot x_{c,i+p,\;j+q,k+r}+b$$

where $w\in R^{C\times P\times Q\times R}$ is the learnable kernel with depth P, height Q, and width R, and b is the bias term. This produces feature maps encoding joint spectral–spatial information, crucial for HSI classification.

The 3DCNN backbone consists of three stages, each combining a 3D convolutional block $C_{i}$ with a subsequent ECA module $\varepsilon_{i}$ (i∈{1,2,3}). Structural details are listed in Table 1.

Stage 1 uses a large spectral kernel (7 × 3 × 3) to aggregate adjacent bands and extract local spectral patterns while suppressing noise, producing 64-channel compact representations. Stage 2 employs a medium spectral kernel (5 × 3 × 3) to capture medium-range dependencies with 128 channels. Stage 3 applies a small spectral kernel (3 × 3 × 3) to model high-level spectral interactions and expands to 256 channels for discriminative mineral feature extraction.

This progressive design—large-to-small spectral kernels, fixed spatial kernels, and expanding channels—captures spectral dependencies from local absorption features to global mineral signatures.

### 3.5. Bidirectional Collaborative Attention Paradigm

The framework establishes a coupled optimization loop between S^3^AM and ECA through gradient backpropagation.

**Forward Pass**: Spatial-Channel Sequential Refinement. The input $X_{in}\in R^{H\times W\times B}$ is first refined by S^3^AM to $X_{1}=A_{s}⊙X_{in}$. It then passes through the three-stage 3DCNN backbone, where each stage is followed by an ECA module, and finally produces ${\tilde{X}}_{3}$.

**Backward Pass:** Gradient-Mediated Bidirectional Coupling. The critical innovation lies in how ECA indirectly modulates the gradient propagated to S^3^AM through the 3DCNN backbone, creating a closed optimization loop. By the chain rule, the gradient of the loss L with respect to S^3^AM parameters $\Theta_{S^{3}AM}$ is:

(16) $$\frac{\partial L}{\partial \Theta_{S^{3}AM}}=\frac{\partial L}{\partial {\tilde{X}}_{3}}\cdot \frac{\partial {\tilde{X}}_{3}}{\partial X_{3}}\cdot \frac{\partial X_{3}}{\partial X_{2}}\cdot \frac{\partial X_{2}}{\partial X_{1}}\cdot \frac{\partial X_{1}}{\partial A_{s}}\cdot \frac{\partial A_{s}}{\partial \Theta_{S^{3}AM}}$$

The key term is the Jacobian of channel recalibration at each stage:

(17) $$\frac{\partial {\tilde{X}}_{i}}{\partial X_{i}}=diag\left(A_{c}^{\left(i\right)}\right)+X_{i}⊙\frac{\partial A_{c}^{\left(i\right)}}{\partial X_{i}}$$

where $A_{c}^{\left(i\right)}$ is the channel attention vector at stage i, and ⊙ denotes element-wise multiplication with broadcasting.

This Jacobian comprises two components:

Direct rescaling term $diag(A_{c}^{\left(i\right)})$: This scales the gradient passing through each channel by its attention weight, amplifying gradients for important channels while suppressing those for redundant ones.

Feature-dependent modulation term $\frac{\partial A_{c}^{\left(i\right)}}{\partial X_{i}}⊙X_{i}$: This captures how changes in feature activations influence the attention weights themselves, creating a feedback loop where feature quality affects attention allocation.

Through this Jacobian, ECA modulates the gradient received by earlier layers. Specifically, the gradient arriving at S^3^AM is a weighted combination.

(18) $$\frac{\partial L}{\partial X_{1}}=(\prod_{i=2}^{3}\frac{\partial X_{i}}{\partial X_{i-1}})\frac{\partial {\tilde{X}}_{3}}{\partial X_{3}}\cdot \frac{\partial L}{\partial {\tilde{X}}_{3}}$$

This bidirectional mechanism is formalized in the joint optimization objective:

(19) $$\underset{\Theta_{S^{3}\mathrm{AM}},\Theta_{3D},\Theta_{\mathrm{ECA}},\Theta_{H}}{\min}E\left[L\left(\hat{y},y\right)\right]+\lambda_{1}R_{S^{3}AM}+\lambda_{2}R_{ECA}$$

where $R_{S^{3}AM}=||1-A_{s}|{|}_{1}$ enforces spatial sparsity for compact focus, encouraging the model to focus on a few key spatial regions, and $R_{ECA}=||A_{c}|{|}_{2}$ ensures stable spectral recalibration by preventing the channel attention weights from becoming too extreme.

Consequently, $A_{c}$ redistributes the gradient received by $A_{s}$, establishing an implicit coupling between spatial masking and channel weighting.

The above design embodies a spatial-first, channel-second collaborative paradigm—a principled departure from conventional attention stacking. In fine-grained hyperspectral mineral classification, spatial interference (e.g., mineral intergrowth, boundary mixing) directly contaminates spectral representations at early stages. Applying channel attention before spatial disambiguation risks amplifying mixed or misleading spectral responses. Therefore, the proposed framework forces a serial dependency: S^3^AM first purifies spatial structures under spectral-similarity guidance; then, and only then, ECA recalibrates spectral channels on spatially refined features. Furthermore, the gradient-mediated feedback from ECA to S^3^AM enables the channel attention to indirectly guide the learning of spatial attention—a closed loop optimization that no parallel or independently stacked attention mechanism can achieve. This design represents a novel collaborative learning strategy beyond conventional attention stacking: it disentangles spatial and spectral ambiguities through ordered, collaborative attention.

**Figure 6:**
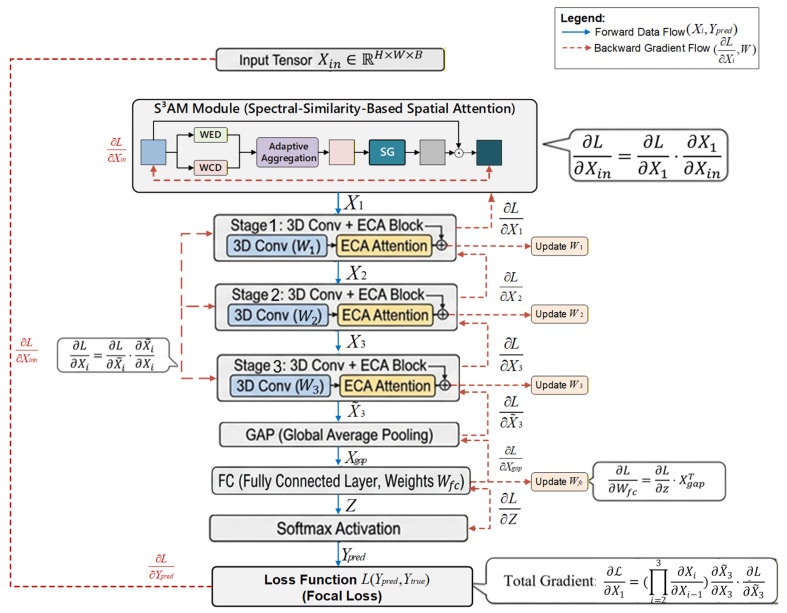
Schematic illustration of gradient flow in the bidirectional collaborative attention paradigm.

This jointly constrained system (illustrated in Figure 6) boosts feature discriminability and robustness against mineral intergrowth and spectral mixing.

Unlike simply stacking S^3^AM and ECA, the proposed framework establishes a spatial-first hierarchical dependency: S^3^AM first suppresses spatially irrelevant responses (e.g., from intergrowth boundaries, shadows, and mixed pixels), after which ECA performs channel-wise recalibration on the purified features. This order respects the intrinsic feature extraction process of hyperspectral mineral imagery—reversing the sequence would amplify noisy spectral responses from mixed or boundary pixels. In contrast to CBAM, which sequentially applies channel then spatial attention via learnable weights, our framework emphasizes a spectral-similarity-guided spatial prior followed by post-encoding lightweight channel recalibration, avoiding generic feature reweighting. The resulting S^3^AM → ECA sequence forms a spatial-first collaborative attention strategy.

### 3.6. Lightweight Classification Head

Following the preceding S^3^AM and ECA stages, the classification head employs an architecture comprising GAP followed by a single FC layer, designed to reduce parameter count and mitigate overfitting, which is particularly important for geological datasets with limited labeled samples per mineral class.

For a 5×5 input patch, the spatial dimensions remain 5×5 after padding; the spectral depth is reduced from 288 to D=35 via three 3D convolutions with strides (2,1,1), paddings (0,0,1) and kernels {7 × 3 × 3, 5 × 3 × 3, 3 × 3 × 3}.

GAP aggregates each channel over its spectral–spatial dimensions:

(20) $$z=GAP\left(X\right)={\left[\frac{1}{D\cdot H\cdot W}\sum_{d=1}^{D}\sum_{i=1}^{H}\sum_{j=1}^{W}X_{c,d,i,j}\right]}_{c=1}^{C}$$

yielding a compact vector:

(21) $$z={\left[z_{1}\;,z_{2}\;,\ldots ,z_{C}\;\right]}^{⊤}\in R^{C}$$

where $z\in R^{256}$ represents the feature vector by removing singleton dimensions.

Specifically, GAP reduces the feature map from [256, 35, 5, 5] to [256, 1, 1, 1], which is reshaped into a 256-dimensional vector and fed into a single FC layer to produce classification logits $\hat{y}\in R^{146}$, corresponding to the number of mineral categories. During training, focal loss [44] emphasizes hard-to-classify samples to handle class imbalance.

Compared to flattening the original feature map, this approach substantially reduces the parameters in the FC layer from approximately 256×35×5×5×146≈32.7M to 256×146≈37.4K, significantly lowering computational complexity while preserving essential channel-wise statistics.

## 4. Discussion

### 4.1. Experimental Design

#### 4.1.1. Datasets

To evaluate the proposed framework under realistic geological conditions, experiments were conducted on a large-scale fine-grained hyperspectral mineral dataset comprising 146 categories, including silicates, carbonates, sulfates, oxides, and sulfides, with substantial diversity in composition, crystal structure, and morphology.

Derived from close-range hyperspectral images of mineral specimens with manually annotated ROIs, the dataset is pixel-based for training and evaluation. Thus, “samples” in Table 2 denote labeled pixels per category, not physical specimens.

Compared with conventional hyperspectral benchmarks, the dataset exhibits pronounced spectral–structural ambiguity, where minerals with different morphologies or crystallization states often present highly similar spectral signatures. Intergrowth and boundary mixing further increase the difficulty of feature discrimination.

Based on manually constructed ground-truth labels, the dataset was divided into training, validation, and test subsets for supervised learning. Table 2 lists per-category pixel counts, revealing class imbalance that reflects natural mineral distributions and offers a challenging benchmark.

#### 4.1.2. Experimental Environment and Evaluation Criteria

All experiments were conducted on an Intel Xeon Platinum 8255C CPU with an NVIDIA GeForce RTX 2080Ti GPU, running Ubuntu 20.04. The implementation used Python 3.8, PyTorch 1.11.0, and CUDA 11.3.

Classification performance was evaluated using three standard metrics: overall accuracy (OA), average accuracy (AA), and Kappa coefficient.

OA is defined as the proportion of correctly classified samples:

(22) $$OA=\frac{\sum_{i=1}^{n}h_{ii}}{\sum_{i=1}^{n}N_{i}}$$

where n is the number of classes, $N_{i}$ is the number of pixels in class i, and $h_{ii}$ is the number of correctly classified pixels in class i.

AA is the average per-class accuracy:

(23) $$AA=\frac{1}{n}\sum_{i=1}^{n}\frac{h_{ii}}{N_{i}}$$

The Kappa coefficient is a consistency metric used to evaluate classification effectiveness, and its formula is given in Equation $\left(24\right)$:

(24) $$Kappa=\frac{N\sum_{i=1}^{r}x_{ii}-\sum_{i=1}^{r}\left(x_{i+}x_{+i}\right)}{N^{2}-\sum_{i=1}^{r}\left(x_{i+}x_{+i}\right)}$$

where N is total samples, r is the number of classes, $x_{i+}$ and $x_{+i}$ are row and column sums, and $x_{ii}$ is the diagonal element.

#### 4.1.3. Experimental Setup

Model weights were initialized using the default PyTorch scheme, with biases set to zero; BatchNorm layers required no additional initialization. The Adam optimizer was used with an initial learning rate of 5 × 10^−4^ and weight decay of 1 × 10^−4^. Learning rate scheduling was performed using ReduceLROnPlateau [45] (factor=0.5, patience=5).

To mitigate overfitting, L2 regularization (weight $decay=1\times 10^{-4}$), gradient clipping, and Dropout3D with a dropout rate of 0.5 were applied. Focal Loss [44] (α=0.25, γ=2.0) was adopted to address class imbalance. Early stopping was implemented by monitoring validation accuracy, with learning rate reduction triggered after 5 epochs without improvement.

A five-fold cross-validation strategy was employed. In each fold, 80% of pixels were used for training, 14% for testing, and 6% for validation. This setup improved generalization evaluation, although variations in sample distribution across folds may have introduced performance fluctuations.

#### 4.1.4. Comparative Experiment of Convolution Kernel Combinations

The spectral progressive downsampling module adopts a three-stage cascaded 3D convolution structure to reduce spectral redundancy while preserving spatial information. Each stage includes BatchNorm, ReLU, and an ECA module for feature enhancement.

A comparative study on kernel size combinations (results are shown in Table 3) shows that, under fixed kernel settings, increasing the spectral kernel size improves performance, indicating the importance of capturing long-range spectral dependencies. However, the best results are achieved with a decreasing multi-scale configuration (7×3×3,5×3×3,3×3×3), outperforming all single-scale designs.

This is because fixed-size kernels cannot simultaneously model spectral features at different ranges. In contrast, the progressive multi-scale structure enables hierarchical feature extraction: the first stage captures long-range dependencies, the second aggregates mid-range information, and the third refines local details. This progressive design better captures spectral variations from broad absorption features to fine-scale details, providing more informative inputs for subsequent collaborative attention paradigm.

To strengthen the comparison of kernel combinations, beyond pairwise *p*-values, we note that although the fixed 7 × 3 × 3 configuration shows no statistical difference from the reference (*p* = 0.815), its much larger standard deviation (5.244% vs. 1.325% for the reference) indicates lower robustness. Computing 95% confidence intervals or applying Bonferroni-corrected paired *t*-tests would more rigorously confirm that the progressive multi-scale design (7 × 3 × 3 + 5 × 3 × 3 + 3 × 3 × 3) achieves not only higher accuracy but also significantly better stability.

#### 4.1.5. Ablation Experiments

To validate the respective roles of S^3^AM and ECA within the proposed framework, a comprehensive set of ablation and comparative experiments was conducted. An improved 3DCNN without any attention mechanism was first adopted as the baseline model and included in all experiments for reference. Attention mechanisms including CBAM [38], SE (Squeeze-and-excitation attention) [46], and SA (Shuffle Attention) [47] were introduced for comparison, alongside variants incorporating S^3^AM or ECA individually. To further investigate the effectiveness of prior-driven versus learnable attention mechanisms, an additional variant was constructed by applying a fixed spectral attention mask based on spectral angle mapping (SAM) [48] to the baseline model. This variant evaluates whether handcrafted spectral-similarity priors can enhance feature discrimination. Moreover, a parallel S^3^AM–ECA–3DCNN architecture was designed, in which spectral–spatial attention (S^3^AM) and channel attention (ECA) are applied independently at the same feature level and fused thereafter, ensuring theoretical consistency while avoiding excessive parameter growth. The classification results are summarized in Table 4.

The baseline is a standard 3DCNN without attention. S^3^AM adds our spectral-similarity-based spatial attention module at the input stage. ECA appends efficient channel attention after each convolutional block. We compare against several attention schemes: SA [47], CBAM [39], SAM (fixed spectral angle mask) [48], S^3^AM+SE (serial S^3^AM followed by SE [46]), and a parallel S^3^AM-ECA-3DCNN where S^3^AM and ECA operate on separate branches and fuse outputs. Our proposed S^3^AM-ECA-3DCNN adopts a serial cascade: S^3^AM first for spatial attention, then the 3DCNN backbone, and ECA after each block for channel refinement.

As shown in Table 4, the proposed serial configuration achieves the highest accuracy. Notably, while the increase in OA is moderate, AA improves more substantially. This is attributed to severe class imbalance in the 146-class dataset (e.g., 5945 vs. 78 pixels per class). Attention mechanisms encourage the model to focus on discriminative spatial–spectral features, benefiting minority classes and boosting AA.

The class imbalance in the HMSD-1.0 dataset is substantial: pixel counts per class range from 78 (Augite, Class 38) to 5945 (Phlogopite, Class 76), a 76-fold difference, with a mean of 3053 and a standard deviation of 1587. Approximately 28% of the 146 classes contain fewer than 1000 pixels, while only 12% exceed 5000 pixels. Under such skewness, conventional models tend to overfit majority classes—inflating OA while providing inadequate representation for minority ones. The pronounced AA improvement observed in our framework confirms that S^3^AM and ECA effectively counteract this bias. S^3^AM suppresses spatial interference from adjacent majority-class pixels, preventing contamination of minority-class spectral signatures, while ECA adaptively recalibrates channel-wise responses to emphasize subtle but diagnostic bands critical for rare mineral phases. This synergistic effect ensures that minority classes receive adequate attention during training, as reflected in the improved AA.

In particular, S^3^AM + SE underperforms compared to S^3^AM alone, likely because SE’s global compression weakens subtle spectral variations among similar mineral classes, whereas ECA preserves local channel interactions and is better suited for fine-grained discrimination.

Parallel S^3^AM-ECA-3DCNN outperforms the baseline but falls short of the serial version. This indicates that parallel application does not fully capture the complementary nature of spatial–spectral and channel attention. The proposed serial architecture’s superiority over individual modules and the parallel variant indicates that the performance gain stems from both the attention mechanisms and, more critically, the explicitly designed spatial-first collaborative dependency. This hierarchical dependency—spatial attention first highlighting informative regions, followed by channel-wise recalibration—aligns better with the progressive feature extraction of 3DCNNs, leading to more discriminative representations and superior performance.

### 4.2. Comparison and Discussion of Experimental Results

To evaluate the effectiveness of the proposed fine-grained mineral classification algorithm, several mainstream multi-class HSI classification algorithms were selected for comparative experiments, including 1DCNN [49], Spec-CNN [50], SPRN (Spectral Partitioning Residual Network) [51], SSRN (Spectral–Spatial Residual Network) [52], S2PST (Spectral structure-aware initialization and probability-consistent self-training) [53], and ACDFSL (Attentive cross-domain few-shot learning) [54]. Table 5 presents the classification results of these models on the 146-category mineral dataset.

Our proposed S^3^AM-ECA-3DCNN achieves the highest OA, AA, and Kappa with the smallest standard deviations, demonstrating superior accuracy and robustness. Among comparative models, ACDFSL approaches our accuracy but lacks stability; Spec-CNN delivers moderate performance; 1DCNN, S2PST, and SSRN show progressively declining accuracy, with S2PST exhibiting large variance; SPRN performs worst across all metrics.

**Figure 7:**
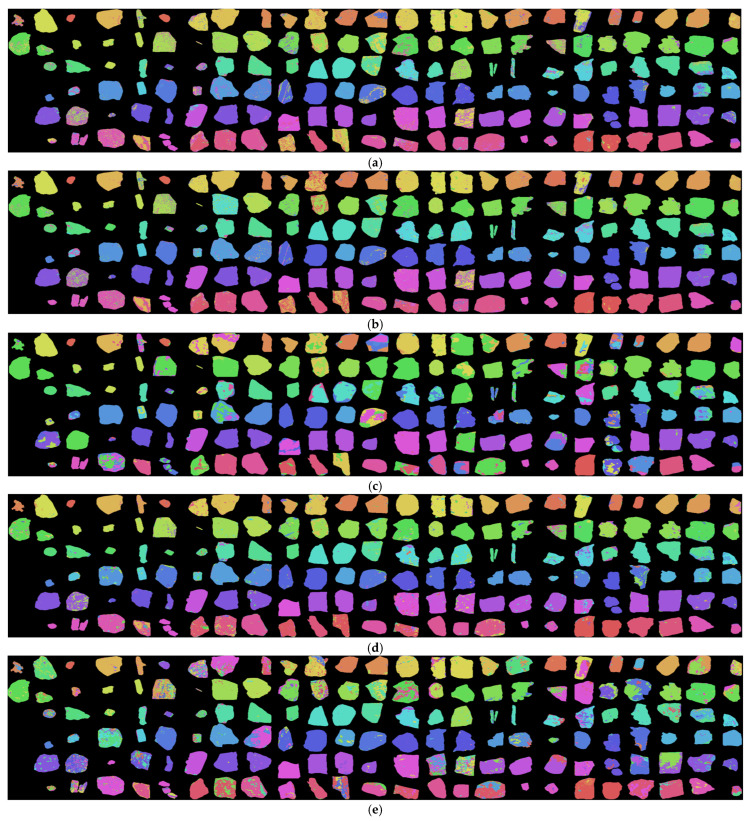

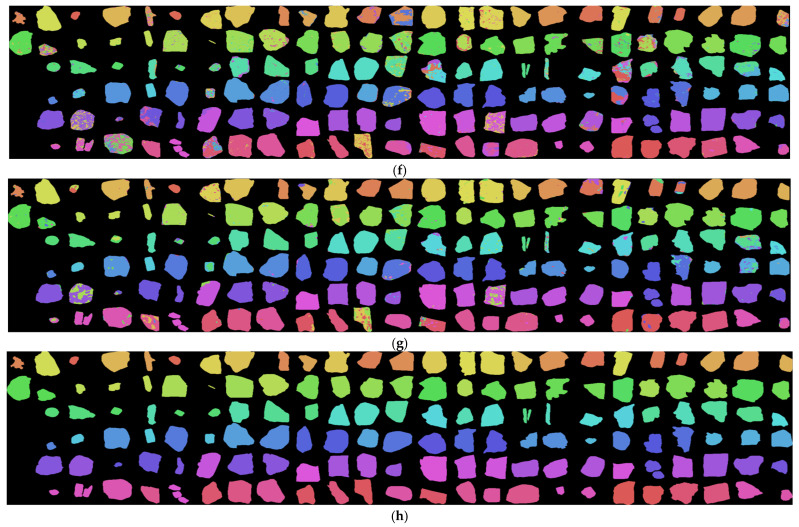
Classification effect of different models on 146 mineral datasets: (**a**) 1DCNN, (**b**) Spec-CNN, (**c**) SPRN, (**d**) SSRN, (**e**) S2PST, (**f**) ACDFSL, (**g**) S^3^AM-ECA-3DCNN, (**h**) true labels after corrosion.

**Figure 8:**
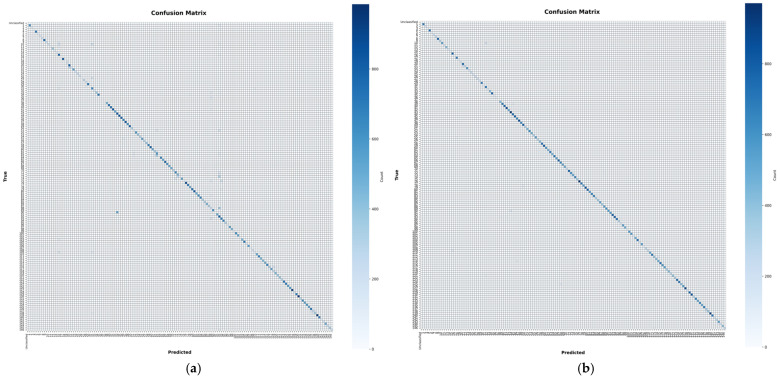

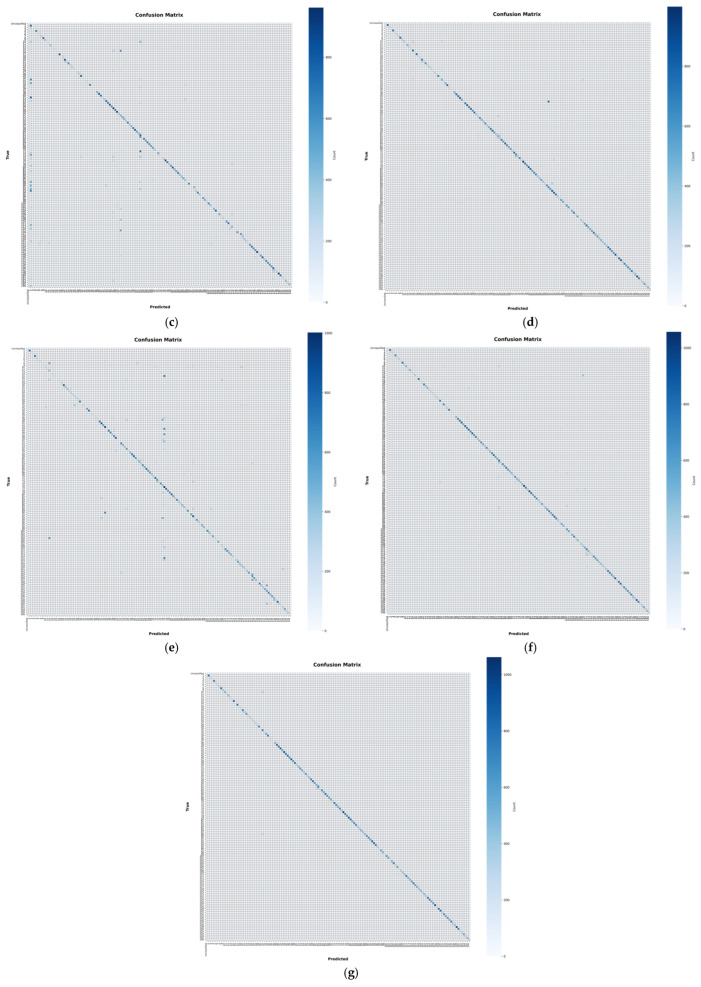
Confusion matrix heat map of different models on 146 mineral datasets: (**a**) 1DCNN, (**b**) Spec-CNN, (**c**) SPRN, (**d**) SSRN, (**e**) S2PST, (**f**) ACDFSL, (**g**) S^3^AM-ECA-3DCNN.

**Figure 9:**
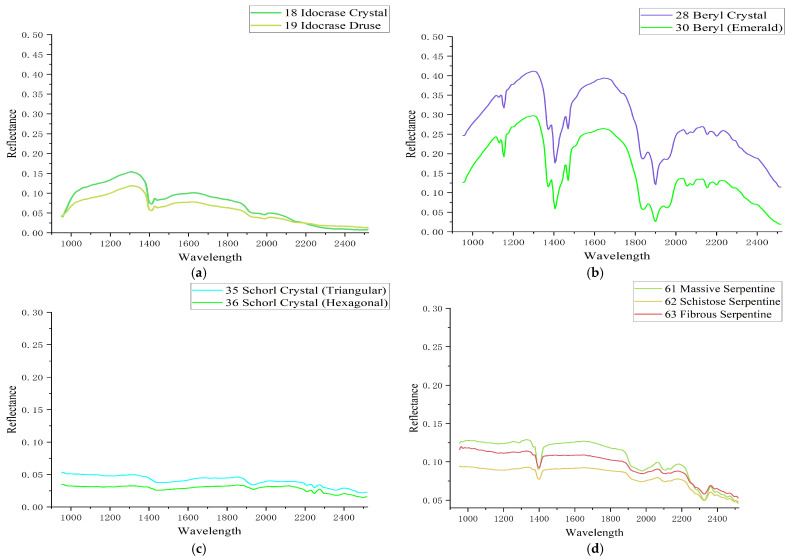

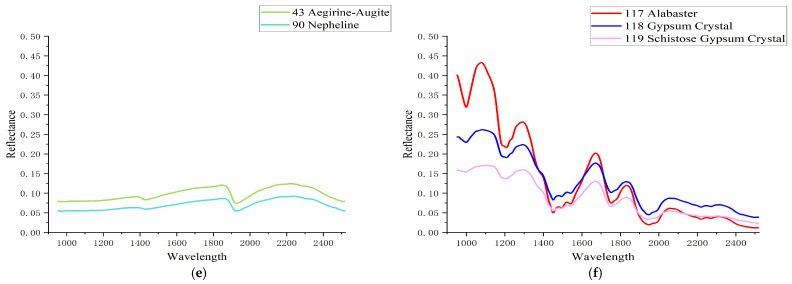
Spectral characteristics of easily confused minerals: (**a**) idocrase (No. 18, green) vs. idocrase crystal (No. 19, pale yellow green); (**b**) beryl crystal (No. 28, violet) vs. beryl (aquamarine; No. 30, green); (**c**) schorl crystal (triangular; No. 35, blue) vs. schorl crystal (hexagonal; No. 36, green); (**d**) massive serpentine (No. 61, green) vs. schistose serpentine (No. 62, mustard) vs. fibrous serpentine (No. 63, red); (**e**) aegirine-augite (No. 36, green) vs. nepheline (No. 90, blue), and (**f**) alabaster (No. 117, red) vs. gypsum crystal (No. 118, blue) vs. schistose gypsum crystal (No. 119, purple).

Figure 7 visualizes classification maps of all seven models, confirming the clear advantage of our method. Confusion analysis of the six baseline models (Figure 8) reveals two systematic failure modes. First, intra-species morphological variations cause high confusion—e.g., idocrase crystal vs. druse (No. 18/19), beryl crystal vs. aquamarine (No. 28/30), schorl triangular vs. hexagonal (No. 35/36), and serpentine varieties (No. 61–63). Second, compositionally identical minerals with different aggregate structures (e.g., alabaster vs. gypsum crystal, No. 117/118) are frequently misclassified. As shown in Figure 9, these conflicting pairs exhibit highly similar spectral signatures despite morphological differences.

Common misclassifications appeared in two scenarios. First, morphological variations within the same species caused high confusion—e.g., idocrase crystal vs. druse (No. 18/19), beryl crystal vs. aquamarine (No. 28/30), schorl triangular vs. hexagonal (No. 35/36), and serpentine varieties (No. 61–63). In addition, mutual misclassification between No. 43: aegirine-augite and No. 90: nepheline, which share similar macroscopic appearances, was frequently observed. The second scenario involved aggregate structures with identical chemical compositions, such as No. 117: alabaster and No. 118: gypsum crystal. These patterns, consistent across all six models, reveal that existing methods poorly handle mineral morphological diversity and structural variability. Figure 9 presents the spectral curves of these easily confused mineral groups, whose spectra remain highly similar despite morphological differences.

To address these fine-grained classification challenges, the S^3^AM-ECA-3DCNN model achieved substantial performance improvements through enhanced feature extraction and structured feature modeling. Experimental results show that the model significantly reduced the major misclassification rates observed in traditional methods. For intra-species morphological variations (e.g., idocrase and schorl), the confusion rate decreased by more than 50% on average compared with 1DCNN and SPRN. For compositionally identical aggregate minerals (e.g., alabaster vs. gypsum crystal), the confusion rate was reduced to 21.05%. In addition, cross-family misclassifications decreased by more than 60% overall.

### 4.3. Computational Complexity Analysis

To further evaluate the computational efficiency of different methods, Table 6 reports the number of trainable parameters, FLOPs, and inference latency. As can be observed, the proposed S3AM-ECA-3DCNN achieves a favorable trade-off between classification performance and computational cost.

All models are evaluated using the same input patch size and hardware platform. The inference latency is measured on an NVIDIA GeForce RTX 2080Ti GPU with a batch size of 64 after 50 warm-up iterations and 100 timed forward passes. The reported latency is normalized by the batch size and represents the average processing time per sample.

As shown in Table 6, the proposed model introduces only a moderate increase in computational cost compared with the baseline network (e.g., 16% higher FLOPs and 15% higher latency), while achieving superior classification performance. Although SPRN contains fewer trainable parameters, its recursive residual spectral convolution strategy results in substantially higher computational complexity and inference latency. In contrast, the proposed S3AM-ECA-3DCNN achieves superior classification performance with only a moderate increase in computational cost over the baseline model, demonstrating a favorable balance between effectiveness and efficiency.

### 4.4. Analysis and Discussion of Misclassification and Imbalanced Dataset

The systematic misclassification patterns of six baseline models—namely, 1DCNN, Spec-CNN, SPRN, SSRN, S2PST, and ACDFSL—are first examined to provide a performance reference for the proposed S^3^AM-ECA-3DCNN. These baselines exhibit pronounced confusion in three distinct scenarios:

1. Morphological variations within the same mineral species: 1DCNN misclassifies 96.4% of idocrase crystal (No. 18) as idocrase druse (No. 19); SPRN misclassifies 97.9% of fibrous serpentine (No. 63) as schistose serpentine (No. 62).
2. Aggregates with identical chemical compositions but different textures: SPRN misclassifies 98.6% of alabaster (No. 117) as gypsum crystal (No. 118), both having the chemical formula CaSO_4_·2H_2_O.
3. Spectral similarity across different mineral families: Owing to the shared Fe^3+^ absorption feature, 1DCNN misclassifies 85.8% of nepheline (No. 90) as aegirine-augite (No. 43).

**Figure 10:**
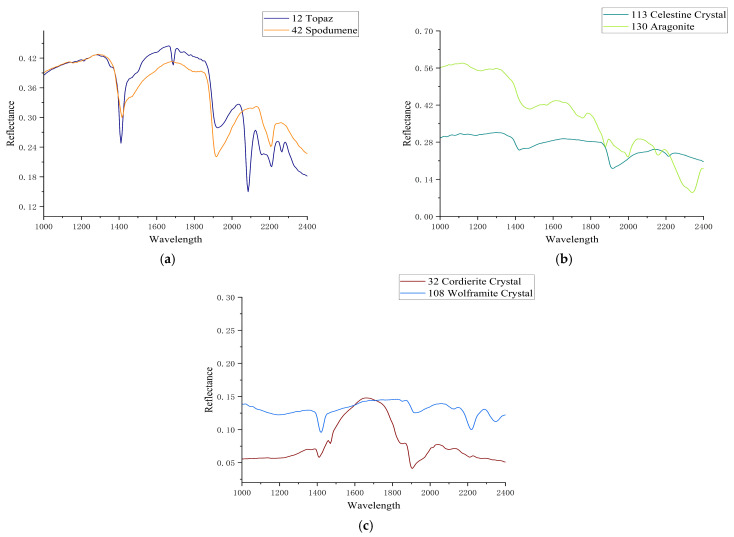
Spectral characteristics of easily confused minerals: (**a**) Topaz (No. 12, dark blue) vs. spodumene (No. 42, orange), (**b**) celestine crystal (No. 113, dark cyan) vs. aragonite (No. 130, green), and (**c**) cordierite crystal (No. 32, wine red) vs. wolframite crystal (No. 108, blue).

Beyond these well-characterized categories, a second tier of persistent misclassifications is observed. Three sub-patterns recur across multiple architectures, as shown in Figure 10:

1. Texture-driven confusion: Talc white (No. 58) is predominantly misclassified as chlorite (No. 80) and wolframite (No. 107) under S^3^AM-ECA-3DCNN (41.8% and 20.5% of misclassified pixels), SPRN (23.5% and 33.5%), and ACDFSL (38.6% and 2.1%). These three minerals show no spectral correlation but share platy/fibrous habits, suggesting spatial attention over-weights textural cues incidentally correlated across morphologically similar, spectrally distinct groups—resulting in a confusion pattern specific to spatial–spectral models.
2. Spectrally driven confusion from overlapping absorption features: Topaz (No. 12) is confused with spodumene (No. 42) under S^3^AM-ECA-3DCNN and ACDFSL, driven by shared spectral features in the visible–NIR range. An analogous mechanism operates for carbonates sharing the CO_3_^2−^ vibrational band near 2340 nm: aragonite (No. 130) is misclassified as celestine crystal (No. 113) under S^3^AM-ECA-3DCNN (37.3%), SPRN (17.3%), SSRN (14.6%), and ACDFSL (18.4%).
3. Confusion from shared heavy-metal absorption edges: Wolframite crystal (No. 108) is systematically misclassified as cordierite crystal (No. 32), reaching 92.2% under SSRN and 27.1% under S^3^AM-ECA-3DCNN, attributable to heavy-metal d–d electronic transitions that suppress diagnostic spectral features.

These three sub-patterns—one texture-driven, two spectrally driven—recur across architectures with distinct inductive biases, revealing the fundamental tension between spatial and spectral representation in hyperspectral mineral classification.

Pronounced class imbalance further compounds these confusion patterns, with per-class sample counts spanning two orders of magnitude (78 to 5945 pixels). While all models exhibit reduced accuracy on lower-sample tiers, sample count alone does not determine classifiability: within the Very Rare tier, stilbite (267 pixels) and staurolite crystal (280 pixels) achieve high accuracy (>99% and 94.1–100%, respectively) owing to their homogeneous crystal habits, whereas apatite single crystal (541 pixels) yields highly variable performance (18.5–99.0%) despite a larger sample. The decisive factor is the coupling of sample scarcity with intra-class morphological diversity—classes encompassing multiple crystal habits or aggregation states constitute an undersampled feature space. This imbalance reflects the natural abundance distribution of minerals rather than any artifact of data acquisition or training protocol. Addressing this challenge will likely require morphology-aware augmentation or few-shot learning paradigms capable of generalizing from limited exemplars across diverse textural appearances.

## 5. Conclusions

This study presents S^3^AM–ECA–3DCNN, a spectral–structural collaborative feature learning framework for fine-grained hyperspectral mineral classification. Unlike conventional hyperspectral classification methods that primarily rely on spectral separability assumptions, the proposed framework is designed to alleviate the challenges caused by spectral–structural ambiguity existing in complex mineralogical scenarios, where minerals associated with different morphologies, aggregate structures, or crystallization states often exhibit highly similar spectral responses at the pixel level.

The framework uses a spatial-first collaborative paradigm: spectral-similarity-guided spatial purification precedes channel-wise spectral recalibration. Experimental results on pixel-level hyperspectral classification demonstrate that this hierarchical attention dependency improves discriminative feature learning under conditions involving mineral intergrowth, spectral mixing, and subtle inter-class spectral variations. Compared with mainstream hyperspectral classification methods, the proposed framework achieves superior classification accuracy, robustness, and generalization capability on a 146-class mineral dataset.

It should be noted that the present study performs pixel-level hyperspectral classification based on manually annotated regions of interest (ROIs). Therefore, the observed improvements in distinguishing minerals associated with different morphologies or aggregate structures are reflected through enhanced pixel-level classification performance rather than through explicit modeling of morphological characteristics.

The experimental results suggest that spatial purification and spectral recalibration function more effectively when jointly optimized rather than independently applied. The bidirectional collaborative mechanism between S^3^AM and ECA enables more stable feature discrimination under spectral homogeneity conditions, providing a new perspective for collaborative spatial–spectral representation learning in hyperspectral image analysis.

Although the proposed framework substantially reduces pixel-level confusion among spectrally similar mineral categories, limitations remain for minerals with nearly identical chemical compositions but distinct morphological characteristics, where spectral information alone may be insufficient for complete discrimination. Future work will explore multimodal feature integration, morphology-aware representation learning, and few-shot adaptation strategies to further enhance fine-grained mineral recognition performance in complex geological environments.

## Figures and Tables

**Table 1:** Structural parameters of the 3DCNN backbone stages.

Stage	Kernel Size ($k_{d}\times k_{s}\times k_{s}$)	Stride (d × h×w)	Padding	Channels (C)
Stage 1	7 × 3 × 3	(2,1,1)	(0,1,1)	64
Stage 2	5 × 3 × 3	(2,1,1)	(0,1,1)	128
Stage 3	3 × 3 × 3	(2,1,1)	(0,1,1)	256

**Table 2:** A total of 146 types of mineral labels corresponding to color and pixel sample number.

Mineral Name		Class	Pixel Count	Mineral Name		Class	Pixel Count
Forsterite	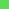	1	4567	Muscovite	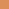	74	3777
Chrysolite	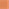	2	984	Biotite	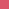	75	2750
Pyrope	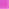	3	434	Phlogopite	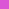	76	5945
Almandine	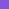	4	4261	Lepidolite	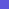	77	4237
Andradite (Demantoid)	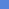	5	548	Zinnwaldite	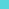	78	3819
Garnet Nodule	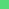	6	1062	Vermiculite	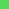	79	5442
Zircon	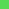	7	1582	Chlorite	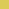	80	4755
Kyanite	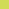	8	4564	Illite	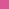	81	3116
Kyanite Crystal	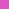	9	1732	Glauconite	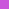	82	4077
Andalusite	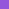	10	4120	Prehnite	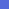	83	3898
Chiastolite	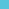	11	962	Orthoclase	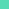	84	2060
Topaz	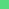	12	2531	Feldspar Crystal	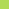	85	2212
Topaz Crystal	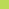	13	737	Microcline (Amazonite)	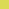	86	3325
Topaz (Placer Deposit)	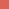	14	583	Plagioclase	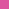	87	2211
Staurolite	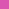	15	5058	Labradorite	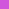	88	3956
Staurolite Crystal	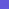	16	280	Leucite	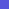	89	3641
Idocrase	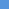	17	4866	Nepheline	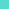	90	4045
Idocrase Crystal	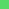	18	648	Sodalite	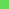	91	3182
Idocrase Druse	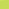	19	769	Nosean	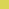	92	5068
Zoisite	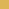	20	4524	Scapolite	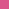	93	5303
Zoisite and Red Corundum Intergrowth	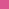	21	2378	Zeolite	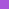	94	3985
Epidote	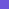	22	3580	Laumontite	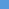	95	1886
Epidote Crystal	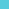	23	1167	Stilbite	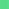	96	267
Epidote and Rock Crystal Intergrowth	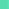	24	1450	Fine-Grained Apatite	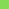	97	497
Allanite	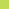	25	1001	Crystalline Apatite	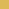	98	2781
Ilmenite	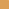	26	1268	Apatite Single Crystal	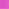	99	541
Beryl	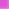	27	523	Apatite Crystal Aggregate	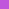	100	3560
Beryl Crystal	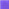	28	935	Pyromorphite	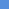	101	3194
Beryl (Emerald)	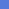	29	1002	Vivianite Crystal	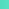	102	835
Beryl (Aquamarine)	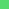	30	4467	Turquoise	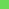	103	3382
Cordierite	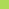	31	427	Turquoise (Spiderweb Turquoise)	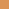	104	4406
Cordierite Crystal	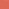	32	4726	Scheelite	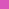	105	1129
Tourmaline (Radiated)	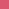	33	768	Scheelite Crystal	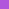	106	3740
Elbaite	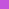	34	3134	Wolframite	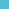	107	486
Schorl Crystal (Triangular)	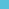	35	4479	Wolframite Crystal	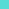	108	1968
Schorl Crystal (Hexagonal)	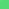	36	878	Barite	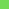	109	2337
Verdelite	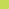	37	748	Blue Barite	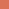	110	3307
Augite	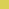	38	78	Barite Crystal	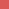	111	4349
Hypersthene	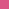	39	3172	Celestine	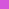	112	2885
Diopside	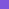	40	4819	Celestine Crystal	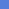	113	2726
Hedenbergite	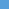	41	4323	Anhydrite	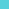	114	3511
Spodumene	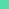	42	5214	Selenite	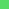	115	4318
Aegirine-Augite	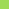	43	3337	Fibrous Gypsum	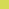	116	3442
Amphibole	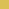	44	5561	Alabaster	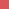	117	3244
Tremolite (Prismatic)	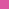	45	4786	Gypsum Crystal	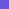	118	1439
Tremolite (Fibrous)	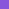	46	4838	Schistose Gypsum Crystal	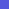	119	725
Cummingtonite	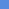	47	3909	Selenite Crystal	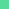	120	2710
Actinolite	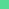	48	5215	Mirabilite	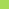	121	2163
Crocidolite	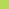	49	3817	Alunite	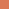	122	2873
Chrysotile Asbestos	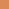	50	4795	Jarosite	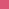	123	2207
Amphibole Asbestos	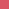	51	1830	Calcite	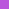	124	4319
Tiger’s Eye (Yellow)	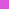	52	2704	laminated calcite	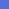	125	4019
Hawk’s Eye (Blue)	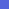	53	3782	Manganoan Calcite	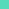	126	3489
Sillimanite	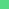	54	3139	Dogtooth Calcite	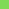	127	3278
Wollastonite	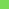	55	1860	Prismatic Calcite	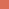	128	5293
Rhodonite	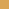	56	4002	Iceland Spar	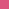	129	1140
Sepiolite	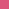	57	1850	Aragonite	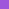	130	4324
Talc (White)	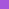	58	2490	Aragonite Crystal	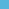	131	5420
Talc (Red)	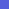	59	4110	Blue Aragonite	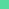	132	1698
Pyrophyllite	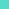	60	4813	Natrolite	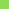	133	3826
Massive Serpentine	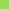	61	3215	Siderite	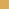	134	3291
Schistose Serpentine	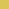	62	3331	White Serpentine Jade	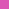	135	3668
Fibrous Serpentine	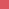	63	4446	Siderite	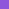	136	3031
Serpentine Jade	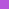	64	3231	Siderite Crystal	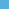	137	4299
Kaolinite	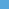	65	4050	Siderite and Stannite Crystal Intergrowth	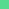	138	2551
Dickite	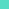	66	3158	Rhodochrosite	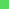	139	3293
Chrysocolla	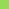	67	4553	Dolomite	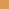	140	5673
Montmorillonite	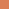	68	3668	Dolomite Crystal	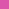	141	4303
Diatomite	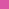	69	3138	Huanghoite	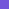	142	1046
Attapulgite	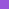	70	3077	Malachite	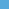	143	1572
Obsidian	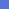	71	1677	Azurite	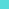	144	3111
Perlite	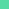	72	4782	Hydrozincite	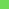	145	1636
Pitchstone	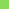	73	3881	Lapis Lazuli	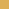	146	2592
Total					445,748		

**Table 3:** Results of multi-scale convolution kernel combination comparative experiment.

Kernel Combination	OA (%)	AA (%)	Kappa × 100	*p*-Value (OA)
3 × 3 × 3 + 3 × 3 × 3 + 3 × 3 × 3	66.875 ± 7.897	58.224 ± 8.488	66.572 ± 7.975	0.004 **
5 × 3 × 3 + 5 × 3 × 3 + 5 × 3 × 3	81.244 ± 0.566	73.818 ± 0.730	81.079 ± 0.571	<0.001 **
7 × 3 × 3 + 7 × 3 × 3 + 7 × 3 × 3	85.277 ± 5.244	80.168 ± 5.887	85.148 ± 5.295	0.815
7 × 3 × 3 + 5 × 3 × 3 + 3 × 3 × 3	85.981 ± 1.325	80.305 ± 2.248	85.859 ± 1.337	—

Note: Results are reported as mean ± standard deviation over multiple runs. *p*-values are computed using paired *t*-tests against the reference configuration (bold row). ** *p* < 0.01 indicates statistically significant difference. The reference combination achieves the highest accuracy with the smallest variance, demonstrating superior robustness.

**Table 4:** Classification results on the 146-class mineral dataset after different modules were added to the baseline.

Models	OA (%)	AA (%)	Kappa × 100
Baseline	85.981 ± 1.325	80.305 ± 2.248	85.859 ± 1.337
S^3^AM	87.523 ± 1.619	82.517 ± 2.147	87.415 ± 1.634
ECA	86.526 ± 0.964	81.249 ± 1.418	86.401 ± 1.005
SA	89.379 ± 6.911	79.652 ± 9.435	89.020 ± 7.038
CBAM	82.417 ± 4.205	76.373 ± 4.872	82.309 ± 4.201
SAM	88.009 ± 1.068	82.803 ± 1.298	87.905 ± 1.078
S^3^AM + SE	86.499 ± 0.921	81.250 ± 1.189	86.381 ± 0.929
Parallel S^3^AM-ECA-3DCNN	91.124 ± 1.107	86.823 ± 1.475	91.050 ± 1.117
Proposed S^3^AM-ECA-3DCNN	93.424 ± 1.398	91.099 ± 2.083	93.368 ± 1.410

Note: Baseline = 3DCNN without attention; S^3^AM = Baseline + S^3^AM at input; ECA = Baseline + ECA after each ConvBlock; SA = Baseline + Shuffle Attention; CBAM = Baseline + CBAM; SAM = Baseline + fixed SAM mask; S^3^AM + SE = serial S^3^AM followed by SE; Parallel = S^3^AM and ECA applied in parallel then fused; Proposed = serial S^3^AM → 3DCNN → ECA (after each block).

**Table 5:** Classification results of different models on 146 mineral datasets.

Models	Architecture	OA (%)	AA (%)	Kappa × 100
1DCNN	1DCNN (spectral-only)	86.136 ± 5.126	83.697 ± 5.504	86.017 ± 5.170
Spec-CNN	1DCNN	89.493 ± 0.194	88.047 ± 0.285	89.403 ± 0.196
SPRN	2DCNN (parallel)	69.392 ± 1.792	66.695 ± 1.959	69.103 ± 1.804
SSRN	3DCNN	84.278 ± 6.425	83.143 ± 6.650	84.822 ± 6.424
S2PST	2D spectral–spatial method	89.369 ± 8.370	88.317 ±8.711	89.276 ± 8.372
ACDFSL	3DCNN	91.644 ± 3.078	90.552 ± 3.367	91.571 ± 3.102
Proposed S^3^AM-ECA-3DCNN	3DCNN + S^3^AM + ECA	93.424 ± 1.398	91.099 ± 2.083	93.368 ± 1.410

**Table 6:** Comparison of model complexity and computational cost among different HSI classification methods.

Model	Params (M)	FLOPs (G)	Latency/Sample (ms)
1DCNN	0.136	0.004	0.0217
Spec-CNN	1.061	0.030	0.0498
SPRN	0.563	5.585	2.7689
SSRN	0.125	1.239	0.516
S2PST	0.134	0.228	0.2328
ACDFSL	1.055	0.446	0.1685
Baseline (3DCNN)	1.040	1.683	0.4083
Proposed S3AM-ECA-3DCNN	1.263	1.951	0.4704

Note: Parameter counts denote the number of trainable parameters. Lower values of FLOPs and latency indicate higher computational efficiency.

## Data Availability

Dataset details and downloads are available at https://uwrl.hznu.edu.cn/c/2023-02-24/2805144.shtml (accessed on 27 August 2024).
